# Manipulation of mRNA translation elongation influences the fragmentation of a biotherapeutic Fc‐fusion protein produced in CHO cells

**DOI:** 10.1002/bit.28230

**Published:** 2022-09-18

**Authors:** Tanya J. Knight, Jane F. Povey, Davide Vito, Atul Mohindra, Colin M. Jaques, C. Mark Smales

**Affiliations:** ^1^ School of Biosciences, Industrial Biotechnology Centre University of Kent Canterbury, Kent UK; ^2^ Biologics Division Lonza Biologcs Slough UK; ^3^ National Institute for Bioprocessing Research and Training Blackrock Co. Dublin Ireland

**Keywords:** Chinese hamster ovary (CHO) cells, clipping, Fc‐fusion protein, fragmentation, mRNA translation elongation

## Abstract

Mammalian cells, particularly Chinese hamster ovary cells, are the dominant system for the production of protein‐based biotherapeutics, however, product degradation, particularly of Fc‐fusion proteins, is sometimes observed that impacts the quality of the protein generated. Here, we identify the site of fragmentation of a model immunoglobulin G1 Fc‐fusion protein, show that the observed clipping and aggregation are decreased by reduced temperature culturing, that the fragmentation/clipping is intracellular, and that reduced clipping at a lower temperature (<37°C) relates to mesenger RNA (mRNA) translation elongation. We subsequently show that reduced fragmentation can be achieved at 37°C by addition of chemical reagents that slow translation elongation. We then modified mRNA translation elongation speeds by designing different transcript sequences for the Fc‐fusion protein based on alternative codon usage and improved the product yield at 37°C, and the ratio of intact to a fragmented product. Our data suggest that rapid elongation results in misfolding that decreases product fidelity, generating a region susceptible to degradation/proteolysis, whilst the slowing of mRNA translation improves the folding, reducing susceptibility to fragmentation. Manipulation of mRNA translation and/or the target Fc‐fusion transcript is, therefore, an approach that can be applied to potentially reduce fragmentation of clipping‐prone Fc‐fusion proteins.

## 1 INTRODUCTION

Immunoglobulin G (IgG)‐Fc fusion proteins represent an important class of biotherapeutic proteins that is often expressed in cultured Chinese hamster ovary (CHO) cell expression systems. These nonnatural molecules can be more difficult to produce than classical IgG molecules with reduced yield compared to a traditional IgG and are susceptible to fragmentation during their production (Dorai et al., 2011; Lim et al., 2018; Robert et al., 2009). Degradation/fragmentation (sometimes also referred to as “clipping”) of the product is undesirable as this can affect the stability and potency of the product but could also generate potentially immunogenic species. Heterogeneity due to degradation can also impact the yield of the molecule obtained and have implications for downstream processing and purification of the undesired fragmented molecule away from the intact, mature molecule required for use in the clinic. To address this, various studies have investigated approaches to enhance the ability of CHO cells to produce higher yields and quality of such molecules (e.g., Bryan et al., 2021; Budge et al., 2020, 2021; Clarke et al., 2019). A number of cellular and mechanical processing steps involved in recombinant protein production have been identified to contribute to the physical and chemical degradation of IgG antibodies (Paborji et al., 1994). Decreased stability of the product has been attributed to aggregation due to increased hydrophobic interactions between unfolded regions (Zhang et al., 2004), or alternatively alterations in physiochemical properties arising from changing pH, product concentration, and ionic strength during downstream purification of the product (Cromwell et al., 2006; Liu et al., 2010; Schreiber, 2002; Telikepalli et al., 2014).

The hinge region of IgGs and Fc‐fusion proteins has been shown to be labile (Cohen et al., 2007; Cordoba et al., 2005; Gao et al., 2011; Gaza‐Bulseco & Liu, 2008; Kamerzell et al., 2010; Vlasak & Ionescu, 2011) with particular regions showing increased susceptibility to hydrolysis or cleavage. This characteristic has been attributed to contributing to the fragmentation of Fc‐fusion molecules during bioprocessing due to the increased exposure of these labile linker regions between the Fc fragment and fusion partner. Direct enzymatic cleavage has also been shown to result in IgG and Fc‐fusion product fragmentation, for example with components of the thioredoxin system shown to bring about antibody reduction (Kao et al., 2010; Koterba et al., 2012; Trexler‐Schmidt et al., 2010), or direct proteolytic attack by host cell proteases (Dorai et al., 2011; Gao et al., 2011; Lim et al., 2018; Mols et al., 2005; Robert et al., 2009; Sandberg et al., 2006; Yang et al., 2019). Host cell protease enzymes are present during, and following the harvest, of the product, and in some cases following downstream purification (Lim et al., 2018; Sandberg et al., 2006; Yang et al., 2019). Indeed, Clarke et al. (2019) used an RNAseq approach to profile cell lines with “high” and “low” IgG4 Fc‐fusion protein clipping or fragmentation and identified protease enzymes that might be responsible for the degradation of the IgG4 Fc‐fusion protein. Six protease genes were identified that were upregulated in high clipping cell lines and then, using protease inhibitors, the authors demonstrated that inhibition of one of these proteases, Furin, resulted in a decrease in clipping of material incubated in conditioned media in the absence of cells.

Clearly, the fragmentation of such Fc‐fusion molecules is undesirable as it leads to increased product heterogeneity, and decreased product quality and can result in the generation of an inactive product. High fragmentation also places increased demand on downstream processes to remove these, which can make a process nonviable. As such, manipulation of process parameters during culture to reduce fragmentation has been investigated. Indeed, a reduction in temperature during culturing has been shown to reduce such fragmentation (Chakrabarti et al., 2016). With regard to reduced temperature culturing, we and others have previously shown that at reduced culture temperature (<37°C) (i) protein synthesis in mammalian cells is reduced and this is impacted via phosphorylation of translation elongation factor eEF2 (Knight et al., 2015; Roobol et al., 2015), (ii) that protein folding can be improved at reduced cell cultivation temperatures (Masterton et al., 2010), and (iii) that defective protein folding and the unfolded protein response (UPR) plays a role in proteolytic clipping of Fc‐fusion proteins (Bryan et al., 2021; Henry et al., 2018). At such reduced culture temperatures, protease expression and activity are also likely to be reduced. Collectively, these studies suggest a link between protein synthesis rates, correct protein folding, Fc‐fusion protein cleavage site accessibility, protease expression, and bioprocess conditions (e.g., temperature, pH, oxygen availability) that govern intact Fc‐fusion protein yields. Here, we describe how the manipulation of mesenger RNA (mRNA) translation impacts the amount of nonfragmented product of a model IgG1 Fc‐fusion protein, showing that manipulation of translation elongation in particular impacts the product quality obtained (quality defined here as intact product vs. fragmented), enhancing the intact to fragmented product ratio obtained from CHO cell expression systems.

## 2 MATERIALS AND METHODS

### 2.1 Cell culture and transfection

CHOK1SV® (Lonza) cell lines were batch cultured using Lonza proprietary conditions with commercial CD‐CHO media (Thermo Fisher Scientific) containing 6 mM glutamine. Stable cell lines were cultured in CD‐CHO media (Invitrogen) containing 25 µM MSX. To induce cold stress, cells were initially incubated with shaking at 36.5°C and 5% CO_2_ for 24 h before switching to 32°C. Transfections were performed using electroporation, BioRad 4 mm cuvettes with 20 µg DNA with 1 × 10^7^ cells using Lonza proprietary methods.

### 2.2 Sodium dodecyl sulphate‐polyacrylamide gel electrophoresis (SDS‐PAGE) and western blot analysis

Supernatant samples were harvested by centrifugation of cultures at 1000 rpm. Cells were lysed in western lysis buffer (10 mM β‐glycerophosphate, 20 mM 4‐(2‐hydroxyethyl)‐1‐piperazineethanesulfonic acid (HEPES), 100 mM NaCl, and 0.5% (w/v) Triton X‐100 with 1 mM NaV, 50 mM NaF supplemented with a protease inhibitor cocktail tablet; Roche cOmplete mini EDTA‐free 11836170001), denatured by addition of Laemmli's buffer and then electrophoresis undertaken after using a Bradford assay to determine equivalent protein loading (for cell lysates). Following transfer to nitrocellulose membranes (GE Healthcare), blots were probed with primary antibodies (see below), horseradish peroxidase‐conjugated secondary antibodies and enhanced chemiluminescence (ECL) (GE Healthcare) was used for detection essentially as previously described in (Roobol et al., 2011). ImageJ (NIH) was used for band quantification. Alternatively, gels were stained using Coomassie blue. Densitometry analysis was undertaken using ImageJ software.

### 2.3 Antibodies

Antibodies were purchased from Sigma‐Aldrich (γ chain [I9764], β‐actin [A5441], rabbit IgG [A6154], mouse IgG [A4416]), Cell Signaling Technology (eEF2 [2332S]), and Santa Cruz Biotechnology (goat‐IgG [SC‐2768]).

### 2.4 Protein A chromatography purification

Protein A purification of cell culture supernatant was performed on an AKTA chromatography system, using a 1 ml HiTrap™ MabSelect SuRe™ column under the control of the Unicorn 5.2 system. Columns were equilibrated and washed using binding buffer (0.018 M sodium phosphate and 0.015 M NaCl, pH 7.2). Elution was performed using 0.1 M sodium citrate, pH 3.5, and elution fractions neutralized using 1 M Tris‐HCl, pH 9.

### 2.5 ^35^S radiolabeling

Cells were seeded at 3 × 10^5^ viable cells/ml in spent media and wells were spiked with 762 kBq of ^35^S radiolabel (TRAN^35^S‐LABEL™; MP Biomedicals, LLC) and incubated at 36.5°C with 5% CO_2_ for 20 h. Following incubation, wells were harvested, and acetone precipitation of the supernatant was performed, and the protein pellet resuspended in radioimmunoprecipitation assay (RIPA) buffer (150 mM NaCl, 1% Nonidet P40, 0.5% sodium deoxycholate, 0.1% SDS, 50 mM HEPES, with a protease inhibitor cocktail tablet; Roche). Cell pellets were either lysed in RIPA buffer or used for a pulse‐chase experiment. The pellet for pulse‐chase was resuspended in fresh media incubated at 36.5°C with 5% CO_2_ for a further 24 h following which the wells were harvested in the same manner as before. Samples could then be analyzed by SDS‐PAGE with further preparation by immunoprecipitation if required. Following Coomassie staining, dried gels were then exposed to ECL hyper film (GE Healthcare).

### 2.6 Immunoprecipitation

Pellet was resuspended in RIPA buffer (150 mM NaCl, 1% Nonidet P40, 0.5% sodium deoxycholate, 0.1% SDS, 50 mM HEPES, with a protease inhibitor cocktail tablet; Roche). Immunoprecipitation was either performed directly with a Protein A Sepharose bead suspension or with prior incubation with primary antibody for 2–4 h on ice. Proteins were then solubilized from the bead pellets by boiling in 2× SDS‐PAGE sample buffer (Invitrogen).

### 2.7 Short hairpin RNA (shRNA) generation

shRNA was produced using the Promega GeneClip™ U1 Hairpin Cloning System. Oligonucleotides were designed from the nucleotide sequence of eEF2 from CHOGENOME™ using a sequence from the start and end of the gene. Hairpin inserts were cloned into the pGeneClip™ Puromycin Vector, then transformed into DH5α *Escherichia coli*. Six colonies were selected for each (start and end targeting shRNA oligonucleotides) and sent for sequencing commercially and four shRNA plasmids were determined to be successful from the sequencing data.

### 2.8 Codon optimization and assessment fragmentation observed from different transcripts

The different transcript‐designed sequences were generated commercially by GeneArt and then cloned into a pcDNA3.1_hygro vector. The Fc‐fusion “standard optimized” was prepared by subcloning from the existing vector into the pcDNA3.1_hygro vector. This sequence was generated using the GeneArt gene optimizer option. Transient transfections were performed via electroporation in duplicate in 10 ml volumes and incubated at 36.5°C at a shaking speed of 220 rpm with 5% CO_2_. Harvest of the cultures was performed at 96 h post‐transfection with individual pellets for RNA extraction and protein analysis prepared at 1 × 10^6^ cells. The supernatant was also collected for analysis of FC‐fusion product levels via SDS‐PAGE and western blot analysis.

## 3 RESULTS

### 3.1 Cleavage of a model IgG1 Fc‐fusion protein occurs at a particular site and appears to be an intracellular event

**Figure 1:**
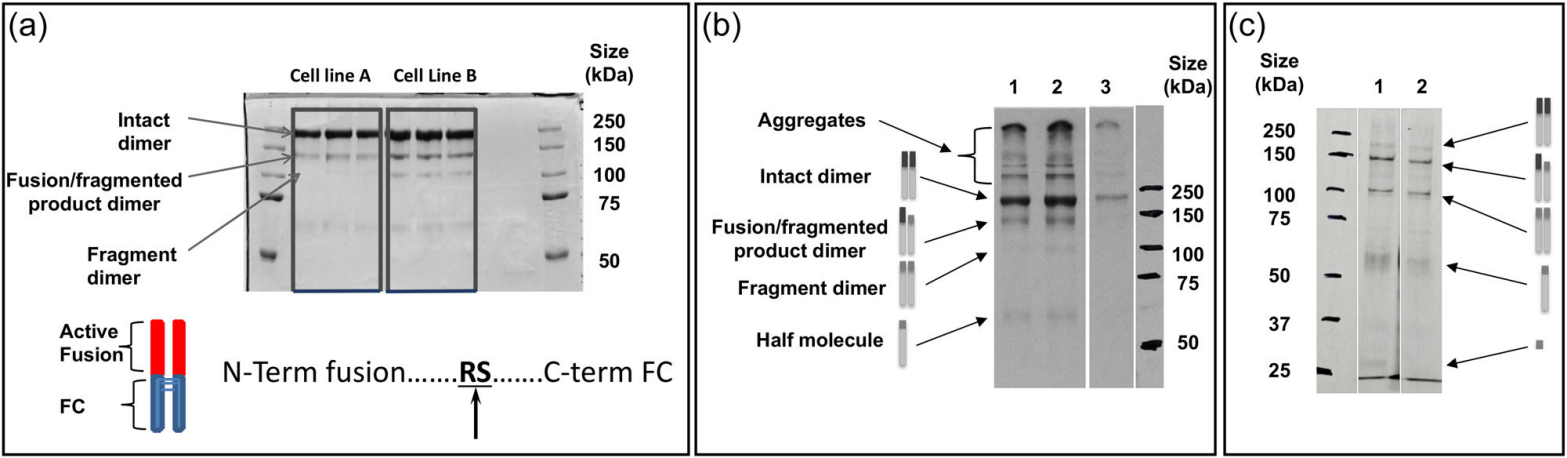
Nonreducing SDS‐PAGE analysis of Protein A‐purified supernatant from two Lonza CHOK1SV® IgG1 Fc‐fusion protein‐expressing cell lines and subsequent determination of cleavage site (a) and ^35^S radiolabelling and pulse‐chase analysis of fusion product species produced from a stable CHO cell line by nonreduced SDS‐PAGE followed by radioautography. (a) Nonreducing SDS‐PAGE analysis of Protein A‐purified material from two IgG1 Fc‐fusion protein expressing CHOK1SV® cell lines and the different species present indicated. The intact product (indicated as intact dimer), single chain fragmented (indicated as fusion/fragmented product dimer) and doubly fragmented (fragment dimer) products were excised from the gel and analyzed by N‐terminal sequencing. This analysis showed cleavage/clipping to occur between an R (Arg) and S (Ser) residue to yield a protein lacking the “active” N‐terminal fusion part of the molecule. (b) Supernatant from stably producing cultures spiked with ^35^S radiolabel to monitor protein synthesis. Protein was precipitated using ice‐cold acetone. Lanes 1 and 2, supernatant precipitated at 20 h after addition of the ^35^S radiolabel to spent media with cells seeded at 0.3 × 10^6^ cells/ml; Lane 3, supernatant precipitated at 44 h after addition of the ^35^S radiolabel (24 h after pulse chase) to spent media with cells seeded at 0.3 × 10^6^ cells/ml. (c) Cell lysate samples from cultures spiked with ^35^S radiolabel to monitor protein synthesis. Samples were immunoprecipitated with antifusion section antibody followed by Protein A Sepharose bead pull‐down. Lane 1, cell lysate at 20 h after addition of the ^35^S radiolabel to spent media with cells seeded at 0.3 × 10^6^ cells/ml; Lane 2, cell lysate at 44 h after addition of the ^35^S radiolabel (24 h after pulse chase) to spent media with cells seeded at 0.3 × 10^6^ cells/ml. In (b) and (c), because the marker proteins are not radioactive, their bands were marked onto the film based on their positions clearly visible on the blot below the film. CHO, Chinese hamster ovary; IgG, immunoglobulin G; SDS‐PAGE, sodium dodecyl sulphate‐polyacrylamide gel electrophoresis.

Initially, we investigated the site of fragmentation in our model IgG1 Fc‐fusion protein and whether the fragmentation of the HC fusion was an intracellular or extracellular event. To determine the fragmentation or clipping site, Protein A‐purified material from the supernatant from two stably expressing CHOK1SV® cell lines was analyzed by nonreducing SDS‐PAGE to reveal the presence of a number of bands (Figure 1a). One of these corresponded to the expected intact IgG1 Fc‐fusion protein, which exists as a dimer of two polypeptides as indicated. The previously observed fragment bands were also present alongside higher molecular weight aggregates that we speculate are due to misfolding and intermolecular disulfide bond formation. The bands of the intact molecule that corresponded to one fragmentation/clipping event and two fragmentation/clipping events in the dimer were then excised and subjected to Edman degradation N‐terminal sequencing. This confirmed the expected N‐terminal was present in the band corresponding to the intact molecule but that in the clipped molecules a new N‐terminal was observed whereby cleavage had occurred after an arginine (R) residue resulting in the loss of 185 amino acids and generation of a new N‐terminal beginning with a serine (S) residue (Figure 1a). This cleavage results in a product lacking the “active” N‐terminal fusion section of the molecule, leaving the Fc portion and being located in the region that links the Fc portion of the molecule to the active fusion partner. The observed clipping after an arginine residue parallels the clipping reported by Clarke et al. (2019) of an IgG4 Fc‐fusion protein whereby three of four fragments identified were clipped after an R residue. However, there was no other sequence similarity in our model IgG1 Fc‐fusion protein and that in the P4–P4′ sequence (where P1 is the Arg residue and those P4–P2 precede this and P1′–P4′ follow) in the Clarke et al. (2019) IgG4 Fc‐fusion molecule.

The study by Clarke et al. (2019) showed that in the absence of protease inhibitors when harvest material supernatant containing the IgG4 Fc‐fusion protein was incubated at 37°C for several days, clipping or fragmentation product continued to accumulate suggesting that more products became fragmented (Clarke et al., 2019). However, incubation of the harvested supernatant of our model IgG1 Fc‐fusion protein for prolonged periods at 37°C showed no increase in the levels of fragmentation observed (data not shown), suggesting either the event causing the fragmentation was an intracellular process or those susceptible molecules were fragmented rapidly upon secretion and hence no additional clipping susceptible molecules remained to be degraded. We, therefore, used a ^35^S radiolabeling approach to follow IgG1 Fc‐fusion polypeptide synthesis intra‐ and extracellularly to determine whether the fragment was derived from the product intra‐ or extracellularly (or both).

To achieve this, one of the CHOK1SV® cell lines used in the data shown in Figure 1a that stably expressed the fusion protein was incubated in the presence of ^35^S Met radiolabel for 20 h after which samples were collected for supernatant analysis. Following this period of incubation, the radiolabel‐containing media was replaced with fresh media (without radiolabel), and the cells were left for a further 24 h at which time cell pellets and culture media samples were again collected. SDS‐PAGE analysis and subsequent radioactivity detection of the cell lysate material and supernatant samples were then performed. Immunoprecipitation was also performed with the cell lysate material to pull down the heavy chain Fc and active component containing material within the cell although we note this approach will not allow detection of any clipped fusion section from the intact molecule. The resulting gel images are shown in Figures 1b,c (and Supporting Information: Figure 1 showing original full autoradiographs with appropriate lanes shown in Figures 1b,c highlighted). Following labeling with ^35^S methionine for 20 h, the major bands observed in the supernatant (Figure 1b) correspond to the intact fusion protein product, alongside fragment species of a molecular weight consistent with the loss of one active component from the dimer (one molecule of Fc‐fusion was cleaved in the dimer) and a second representing the loss of both active components. A further band at around 60 kDa was observed that corresponds to the heavy chain Fc fragment with some of the linker and fusion partners as predicted from the identified cleavage site (labeled half‐molecule in Figure 1b,c). Quantitation by densitometry of the bands observed is reported in Supporting Information: Figure 1. The fragmented protein was observed in the supernatant after the initial labeling period of 20 h and after a further 24 h, although the amount of intact to the fragmented product in the supernatant did not appear to increase suggesting that further fragmentation of material was not observed over time. This supports the hypothesis that a proportion of the material produced is susceptible to fragmentation and not that all material is susceptible, and that which is susceptible is clipped rapidly.

Figure 1c shows cell lysate samples from the radiolabeling treatment, which were immunoprecipitated with antibodies targeting the active Fusion component of the molecule to determine if clipping occurred intracellularly and if the cleaved fragment could be identified intracellularly. Once again, a number of species were present, including those that apparently do not contain the fusion component of the molecule following pull‐down. This is explained by the fact that the Protein A pull‐down will directly pull down the HC component of the Fc‐fusion protein, as well as the anti‐fusion antibody bound to the fusion section, inadvertently resulting in pull‐down of all species. This approach showed the clipped products were present intracellularly and appeared to be at higher amounts than the intact product (confirmed by densitometry analysis, Supporting Information: Figure 1), suggesting some of the fragmented product may be retained within the cell, whereas the fully folded and assembled intact protein is secreted. A further band was observed in the cell lysate samples after the 20‐h incubation with the ^35^S radiolabel that was not observed in the supernatant analyses at approximately 26 kDa which corresponds to the expected size of the cleaved active fusion fragment (Figure 1c). Collectively, these data support the hypothesis that the fragmentation event mainly occurs intracellularly, before the molecule is secreted. This is in agreement with the hypothesis that species susceptible to fragmentation may be the result of folding issues (Henry et al., 2018) which leaves regions accessible to proteases and fragmentation.

### 3.2 Addition of protease inhibitors to the culture supernatant does not prevent the generation of fragmented products

**Figure 2:**
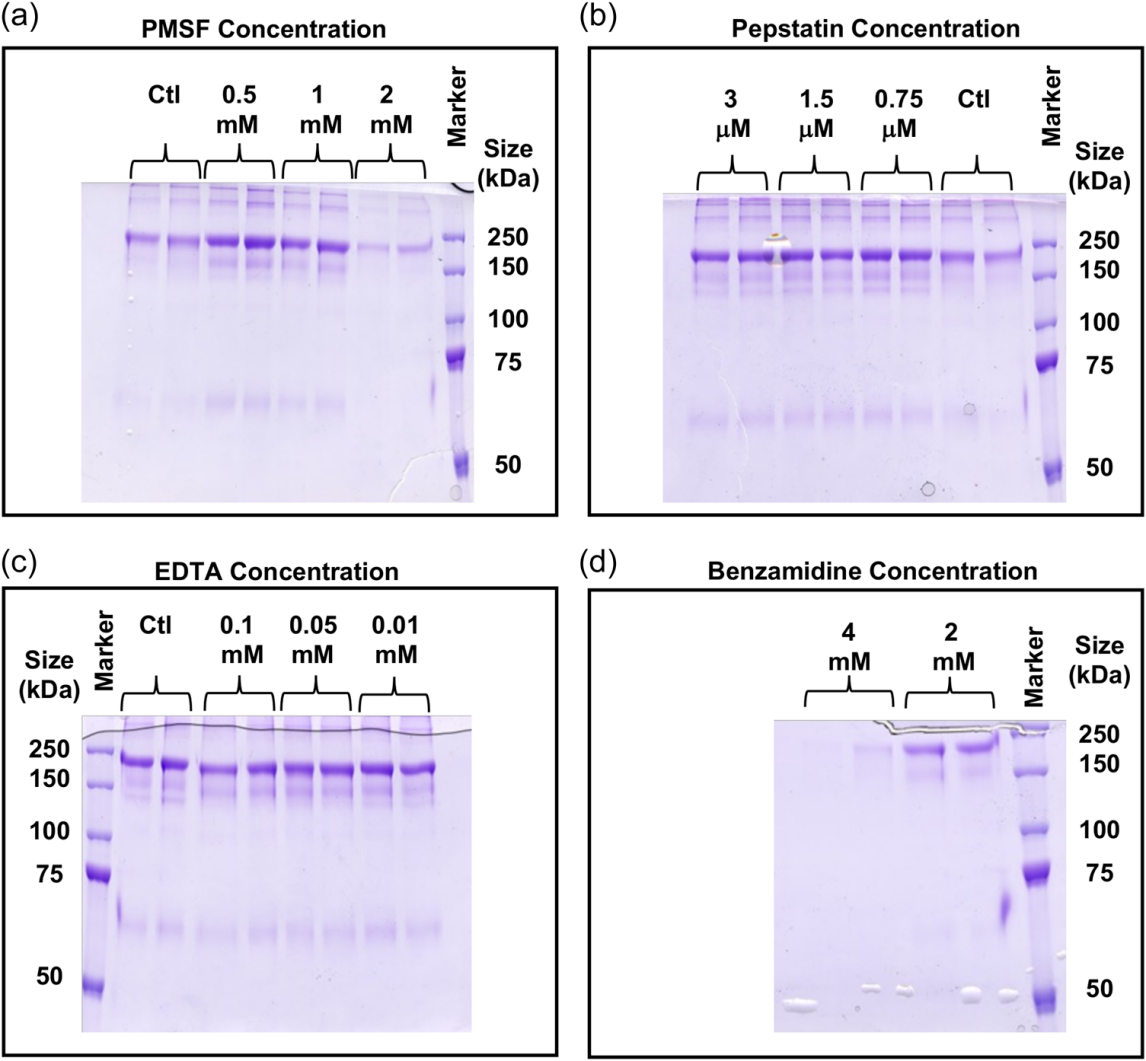
Addition of protease inhibitors to a stable CHOK1SV® producing IgG1 HC Fc‐fusion cell line and the impact on fragmentation. Various classes of protease inhibitor were incubated with a cell line stably expressing the model IgG1 HC Fc‐fusion protein for 6 days in bath culture, adding the concentration of inhibitor indicated at Day 0 and again after 3 days. Supernatant samples were harvested after 6 days of culture, and the product purified using Protein A chromatography and analyzed by nonreducing SDS‐PAGE. (a) Addition of 0.5, 1, and 2 mM PMSF or ethanol control. (b) addition of 0.75, 1.5, and 3 µM pepstatin or ethanol‐treated control. (c) addition of 0.01, 0.05, and 0.1 mM EDTA or control. (d) addition of 2 and 4 mM benzamidine. Staining was performed with Coomassie brilliant blue G250. CHO, Chinese hamster ovary; PMSF, phenylmethanesulfonyl fluoride; SDS‐PAGE, sodium dodecyl sulphate‐polyacrylamide gel electrophoresis.

We next investigated whether addition of protease inhibitors to cell culture during batch culture would reduce fragmentation. Clarke et al. (2019) showed that addition of a protease inhibitor for a specific serine endoprotease could prevent further fragmentation or clipping of an IgG4 Fc‐fusion protein in cell‐free culture supernatant. Analysis of the fragmentation site for our model IgG1 Fc‐Fusion protein in the MEROPS peptidase database (Rawlings et al., 2014) suggested that serine proteases were a potential protease class that could be responsible for fragmentation at the site identified, although cysteine and metalloproteases were also highlighted as possibly being able to cleave the amino acid sequence. To investigate these possibilities further, the levels of fragmentation were determined following batch culture of a CHOK1SV® cell line stably expressing the IgG1 HC Fc‐fusion product in the presence of protease inhibitors. We particularly investigated the addition of phenylmethanesulfonyl fluoride (PMSF), an inhibitor of serine proteases as well as cysteine proteases; benzamidine hydrochloride, an inhibitor of trypsin and trypsin‐like enzymes; Leupeptin hemisulfate: a reversible inhibitor of trypsin‐like proteases and cysteine proteases; Pepstatin, an aspartic protease inhibitor, and EDTA, a metalloprotease inhibitor. The concentrations of the inhibitors used were selected based on those previously shown in the literature to have a beneficial effect when added to culture postharvest (e.g., Cordoba et al., 2005; Dorai et al., 2011; Sandberg et al., 2006). Incubation with the protease inhibitors resulted in no obvious and defined change in the fragmentation pattern or ratio of intact to fragmented product (Figure 2). All concentrations investigated showed comparable or increased levels of fragmentation compared to the control groups (Figure 2). A reduced amount of fragment was observed with 2 mM PMSF and 4 mM benzamidine, but this was also accompanied by a decrease in intact product and was a reflection of incubation with the protease inhibitor affecting cell growth and culture viability (data not shown). A possible reason that the protease inhibitors investigated here did not result in a decrease in the observed fragmentation is that the inhibitors were unable to cross the cell membrane and the product, which is susceptible to intracellular fragmentation was, therefore, already cleaved before secretion. We also investigated combinations of inhibitors and a wider range of inhibitors but none of these approaches showed any impact on fragmentation (data not shown).

### 3.3 Reduced temperature cultivation results in a reduced fragmentation profile of the model IgG1 Fc‐fusion protein

**Figure 3:**
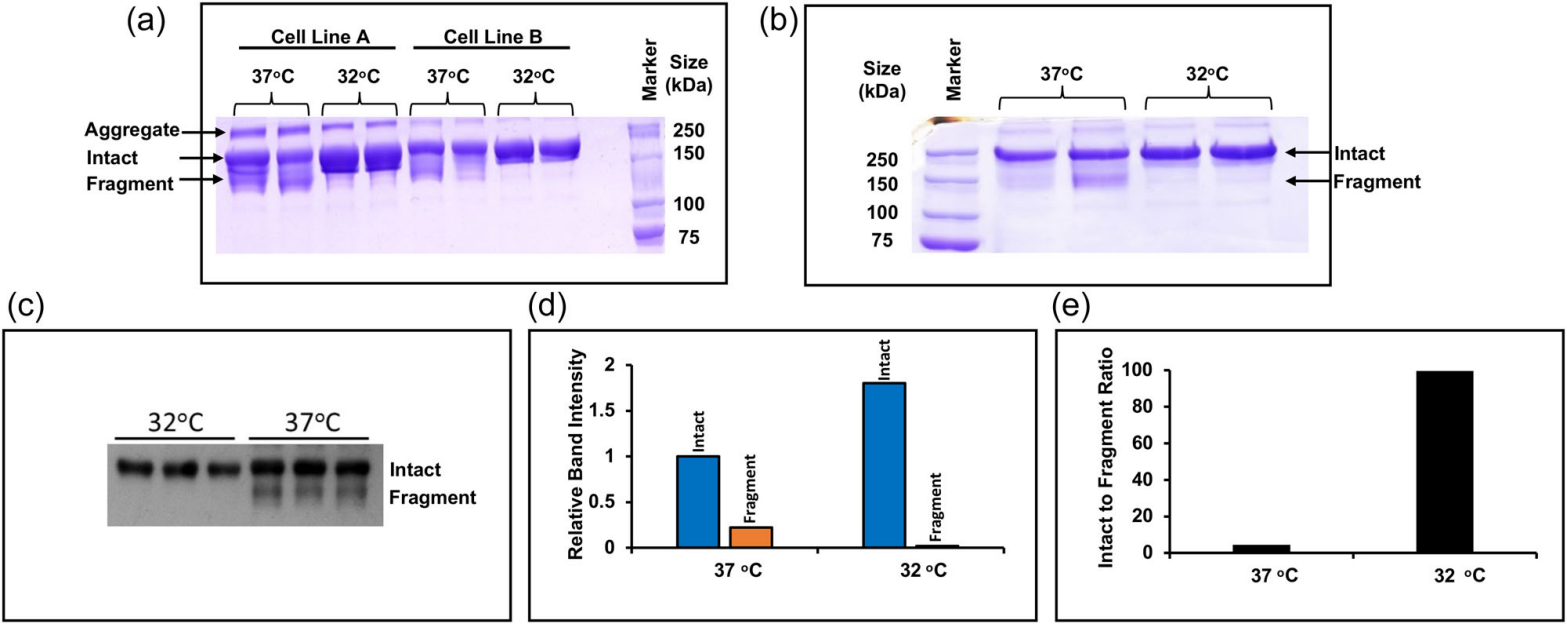
A temperature shift to 32°C during culture reduces IgG1 HC Fc‐fusion molecule fragmentation levels. (a) Supernatant from IgG1 HC Fc‐fusion stably expressing cell lines A and B were cultured at either 37 or 32°C for 7 days. At the end of incubation, supernatant from the cultures was harvested, purified by Protein A chromatography, and analyzed by nonreducing SDS‐PAGE followed by visualization of the bands present by Coomassie blue staining. The image shows supernatant samples from Day 7 of culture. (b) SDS‐PAGE analysis of the Protein A‐purified product from cell line A after a 7 day batch culture at 32°C. (c) Supernatant samples from HC Fc‐fusion stably expressing cell line A cultured at either 32°C or 37°C. Supernatant was harvested on Day 4 of culture and analyzed by nonreducing SDS‐PAGE followed by western blot analysis. Western blots were probed with anti‐HC antibody. (d) Densitometry analysis of the average band intensity of the intact or fragment bands at 37 or 32°C in (b). (e) The intact to fragment band intensity ratio calculated from the data in (d). IgG, immunoglobulin G; SDS‐PAGE, sodium dodecyl sulphate‐polyacrylamide gel electrophoresis.

Addition of protease inhibitors to culture or during downstream processing, even if shown to successfully reduce fragmentation, is not a desirable solution for manufacturing as inhibitors are expensive, they must be removed during downstream processing, and may require bioprocess redesign so that they can be added at appropriate times during a process. As a shift to lower culture temperatures (<37°C) has previously been reported to reduce fusion protein fragmentation (Chakrabarti et al., 2016) we used this approach to investigate the impact of reduced temperature batch culture on the model IgG1 Fc‐fusion protein. We note that reduced temperature cultivation also results in slowed mRNA translation elongation during protein synthesis (Knight et al., 2015). We batch cultured two CHOK1SV® cell lines expressing the model Fc‐fusion protein at either 37°C or 32°C for 7 days and then purified the material in the cell culture supernatant by Protein A chromatography and analyzed the fragmentation pattern by nonreducing SDS‐PAGE and western blot. A decrease in the amount of fragmentation in the two different stably expressing cell lines was observed after 7 days of batch culture alongside a corresponding increase in the levels of intact product at 32°C compared to when cultured at 37°C (Figure 3a). There was also a reduction in the amount of the larger molecular weight aggregate species observed at 32°C (Figure 3a). To quantitate the difference in the intact product and fragmented material produced, material from a Day 7 batch culture of cell line A at either 37°C or 32°C was harvested and then analyzed by SDS‐PAGE (Figure 3b). The fragment was more clearly resolved in this gel (Figure 3b). Subsequent densitometry analysis of the intact and fragment bands revealed that not only was the amount of intact material produced increased at 32°C compared to 37°C (i.e., a higher yield despite lower cell numbers at 32°C) but that the amount of fragment observed was reduced too (Figure 3d). As a result, the ratio of intact to fragment material produced was massively increased (Figure 3e). Therefore, reduction of culture temperature not only reduced fragmentation resulting in increased yield of the desired product but further reduced heterogeneity by reducing the amounts of the higher molecular weight species. When we used western blot analysis to analyze samples taken from one cell line on Day 4 of the culture, again a reduction in the amount of fragmented product was observed at 32°C compared to 37°C (Figure 3c), showing that fragmented product was produced at least across Days 4–7 of culture at 37°C and was not associated with late batch culture samples when culture viability began to decrease.

### 3.4 The beneficial effects of reduced temperature culture on Fc‐fusion protein fragmentation can be mimicked by directly influencing and slowing mRNA translation elongation at 37°C

Reduced temperature cultivation decreased Fc‐fusion protein fragmentation in agreement with other reports that a reduction in temperature alters the metabolism and improves the sustainability and fidelity of recombinant protein‐producing cell lines (Masterton & Smales, 2014; Masterton et al., 2010; Roobol et al., 2020). Such subphysiological temperatures slow translation by influencing a number of cellular responses, including the phosphorylation of the translation elongation factor eEF2, resulting in the slowing of polypeptide elongation (Knight et al., 2015; Proud, 2007). We, therefore, sought to mimic the effect of reduced temperature culturing at 37°C, without reducing the temperature, by slowing translation elongation to determine if manipulation of polypeptide elongation influenced the Fc‐fusion protein fragmentation pattern. To achieve this we used cycloheximide, an established chemical agent that inhibits eukaryotic translation elongation (Schneider‐Poetsch et al., 2010). Cycloheximide was added to a CHO cell line expressing the IgG1 Fc‐fusion molecule and the effect on fragmentation levels was determined. The concentrations of cycloheximide added to cultures were based on those previously shown in CHO cells to give a 30–40% reduction in protein synthesis (Rossow et al., 1979; Traganos et al., 1987). This level of reduction in protein synthesis is in line with that observed upon temperature downshift to 32°C (Knight et al., 2015) and was, therefore, considered to be the “equivalent” of temperature reduction in terms of the effect on global protein synthesis.

**Figure 4:**
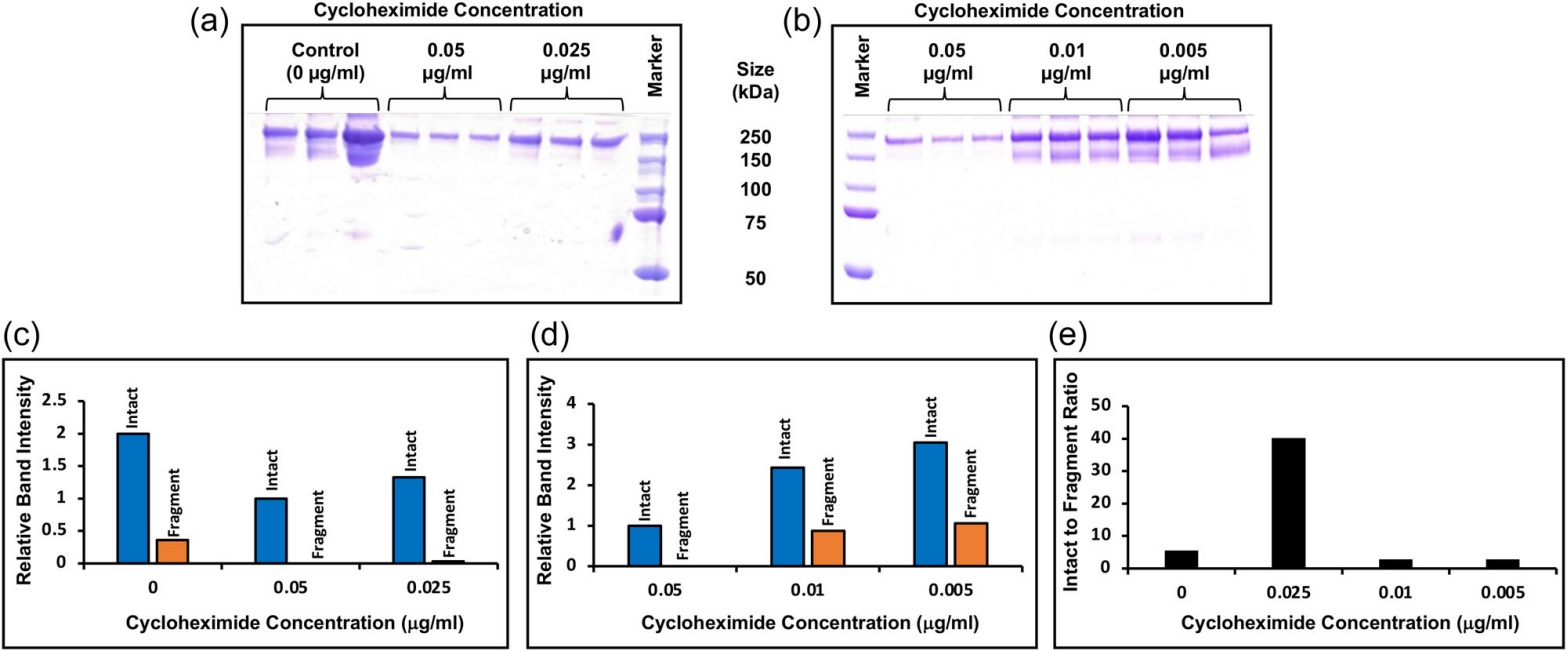
Slowing of translation elongation by the addition of the chemical inhibitor cycloheximide to cultures of CHOK1SV cells stably expressing a model IgG1 Fc‐fusion molecule results in reduced fragmentation. Cultures of the cell line A stably expressing the fusion protein, incubated with (a) 0 (control), 0.05, 0.025 and (b) 0.05, 0.01, and 0.005 µg/ml cycloheximide. All concentrations were investigated in triplicate. Cultures were harvested after 7 days and the supernatant was purified using Protein A chromatography, the elution fractions were concentrated and analyzed on an 8% nonreduced SDS‐PAGE with Coomassie staining. (c, d) Densitometry analysis of the average band intensity of the intact or fragment bands in (a, b). (e) The intact to fragment band intensity ratio calculated from the data in (c, d) (note as there is no fragment detected at 0.05 µg/ml cycloheximide there is no data for this concentration). IgG, immunoglobulin G; SDS‐PAGE, sodium dodecyl sulphate‐polyacrylamide gel electrophoresis.

The slowing of mRNA translation, and hence polypeptide elongation, upon the addition of cycloheximide, resulted in a reduction in the levels of fragmentation of the fusion molecule observed at a concentration of 0.05 and 0.025 µg/ml (Figure 4). At the highest concentration used (0.05 µg/ml) there was a reduction in fragmentation but also of unclipped (intact) material, thus, slowing of translation elongation by chemical methods reproduced the effect of a temperature shift to 32°C in terms of reducing fragmentation and provides evidence that the effect of reduced fragmentation at 32°C is mediated via slowed translation elongation, and not simply due to reduced protease activity. However, an increase in intact product yield over the control was not observed. The decreased fragmentation is likely the result of improved folding at slower protein synthesis rates and, thus, reduced access of the cleavage site to proteolysis, although this was not confirmed here.

### 3.5 Can direct manipulation of eEF2 amounts reduce Fc‐fusion protein fragmentation?

**Figure 5:**
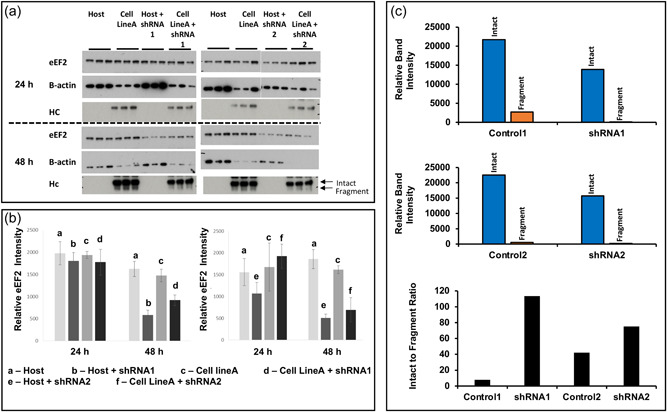
The impact on Fc‐fusion protein fragmentation via direct manipulation of eEF2 amounts using shRNA. Stably IgG1 Fc‐fusion protein‐producing cell line A and the host cell line were transfected with shRNAs designed to the CHO cell genome eEF2 sequence using the GeneCLIP system. Supernatant and cell lysate samples were taken 24 and 48 h after transfection. Western blot analysis was performed using an anti‐eEF2 antibody (Cell Signalling) with β‐actin as a loading control. Supernatant was analyzed by western blot analysis using an anti‐HC antibody (a). (b) Densitometry analysis was performed using ImageJ software to confirm eEF2 knockdown. (c) Densitometry analysis of the average band intensity of the intact or fragment bands, as indicated in (a) for the 48 h time point and the calculated intact to fragment band ratio. CHO, Chinese hamster ovary; eEF2, elongation factor 2; IgG, immunoglobulin G; shRNA, short hairpin RNA.

As we had shown that slowing mRNA translation, specifically elongation either using chemical agents such as cycloheximide or by reducing culture temperature, results in a reduction in the levels of fragmentation of the Fc‐fusion protein molecule, we investigated if direct manipulation of eEF2 amounts could achieve the same impact on fragmentation that the chemical or temperature methods did. Previous reports have shown that knockdown of elongation factor 2 (eEF2) suppresses protein synthesis (Shmookler et al., 2021). shRNAs were, therefore, generated to knockdown CHO eEF2 using the GeneClip system targeted to different regions of the eEF2 sequence. The resulting plasmids were transfected into a cell line stably producing the Fc‐fusion molecule and the nonproducing CHOK1SV® host cell line. Apparently reduced levels of the eEF2 protein were detected in the presence of the shRNA after transfection in both cell lines (Figure 5). Both shRNA constructs showed a more pronounced effect 48 h after transfection on eEF2 expression, with a 64% and 73% reduction in eEF2 protein achieved with shRNA 1 and 2, respectively in the host cell line. Lower reduction of eEF2 expression (38% and 57%) was achieved with shRNA 1 and 2, respectively, after 48 h in the stably producing Fc‐fusion cell line (Figure 5b). Generally, protein synthesis will be reduced upon eEF2 knockdown as confirmed by a reduction in β‐actin signal in cell lysate samples at 48 h compared to 24 h (Figure 5). Despite this, Fc‐fusion protein fragmentation was reduced at 48 h post‐transfection upon knockdown of eEF2 compared to controls. This may, at least partly, reflect the lower overall levels of Fc‐fusion protein made when protein synthesis is reduced, however, the data provides further evidence that the slowing of translation elongation, in this case through the direct manipulation of eEF2 levels, reduces Fc‐fusion molecule fragmentation. In agreement with the cycloheximide experiments, fragmentation was substantially reduced upon knockdown as shown by the intact to fragment ratio of the knockdowns versus controls but overall intact material was reduced by approximately 30%, again in line with the cycloheximide elongation inhibitor data (Figure 5c).

### 3.6 Altering the codon usage in the Fc‐fusion sequence can be used to tune its expression and fragmentation profile

Manipulation of global protein synthesis by reducing eEF2 levels resulted in decreased viable cell concentrations and culture viabilities as would be expected. Therefore, it would be desirable to impact Fc‐fusion product‐specific translation speeds without impacting the overall translational capacity of the cell. Codon optimization is routinely performed to design recombinant mRNA sequences for the most abundant codons used in the host species in an attempt to enable the most efficient translation speeds for the product. However, this does not take into account the predicted translation speeds of a sequence and the available transfer RNA (tRNA) population for subsequent translation of the mRNA. Therefore Fc‐fusion mRNA nucleotide sequences of our model were designed based on predicted elongation rates for the sequence based on a eukaryotic translation model (Chu et al., 2014) and tRNA abundance for Chinese hamster. Sequences were designed to give transcripts that would be decoded (elongated) at different speeds and, thus, have faster or slower protein synthesis rates.

**Figure 6:**
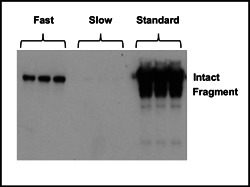
Determining the impact on fragmentation of manipulation of translation rates via messenger RNA sequence. The CHO host cell line was transiently transfected using electroporation with sequences designed via standard commercial codon optimization (standard) or using a balance of fast (fast) or deoptimized (slow) codons in the translation model developed by Chu et al. (2014) and using Chinese hamster tRNA abundances. Supernatant samples were harvested 96 h after transfection and assessed for recombinant product levels via western blot analysis using an anti‐HC antibody (Sigma‐Aldrich). CHO, Chinese hamster ovary; tRNA, transfer RNA.

Following transfection and transient expression for 96 h in host CHOK1SV® cells, the fusion protein was detectable in those cultures transfected with two of the three sequences (Figure 6). As expected, the disoptimized (slow, using the slowest decoded codons as predicted by the model) sequence showed the lowest yield with little to no observable expression. The levels of expression differed markedly between the sequence optimized using “standard” commercial codon optimization for the most abundant codons compared to that using the Chu model and tRNA abundance. The “standard” optimized sequence gave the highest Fc‐fusion protein expression but also had large amounts of the fragmented species. There was less protein expressed from the transcript designed with the Chu model using the fast sequence (fastest decoded codons), but only a band corresponding to the full, intact protein was observed and no detectable fragmentation. This confirms that the codon usage and subsequent speed of protein synthesis can be used to not only manipulate protein expression but also be used to tune secreted intact‐fragment ratios and yields. We cannot rule out that intracellular retention could account for the reduced amount of material observed in the supernatant samples of the “fast” sequence or that there were impacts on other measures of protein quality in addition to fragmentation such as glycosylation, folding, or activity. The advantage of this approach is it does not require the addition of reagents to the culture to reduce or eliminate fragmentation.

## 4 DISCUSSION

Here, we show that for a model IgG1 Fc‐fusion protein prone to fragmentation (or clipping) that mRNA translation elongation and protein synthesis is related to the amount of fragmentation observed. Our data collectively shows that there is a balance between the speed at which the protein is produced, and the amount of fragmentation observed. Our investigations show that for this particular molecule, fragmentation of the Fc‐fusion molecule appears to occur soon after synthesis, intracellularly. Both the intact and fragmented species were identified intracellularly and in the cell culture supernatant after 20 h of ^35^S radiolabeling. Pull down with an antibody targeting the fusion protein active component of the Fc‐fusion molecule identified a band of the molecular weight consistent with the size of the active component intracellularly following fragmentation. This is in contrast to the study by Clarke et al. (2019) on a model IgG4 Fc‐fusion protein that was shown to continue clipping extracellularly in cell‐free conditioned media (Clarke et al., 2019). Collectively our data provide evidence that the fragmentation of the IgG1 Fc‐fusion protein investigated here occurs intracellularly with a proportion of the product appearing susceptible to the fragmentation event. Further, in comparison to the study by Clarke et al. (2019), our studies show that different Fc‐fusion proteins are likely to fragment via different mechanisms and be clipped by different proteases that may be present intracellularly or extracellularly due to secretion or release during cell lysis/death.

Our data shows that the slowing of mRNA translation, predominantly through reduced translation elongation, has a beneficial effect on the quality of Fc‐fusion product produced in that less fragmented material is produced. This was demonstrated using sub‐physiological temperature culturing, the use of chemical agents to stall elongation, as well as the manipulation of the codon sequence to slow the speed of translation elongation. The benefits of temperature shift for recombinant protein production have been documented in the literature (see Masterton & Smales, 2014 for review) and translation speed, specifically elongation has been shown to influence polypeptide folding and levels of cotranslational ubiquitination observed (Sherman & Qian, 2013). The ability to reduce aggregation of an Fc‐fusion molecule has also been achieved previously by influencing translation initiation and upregulation of PERK via subphysiological culture temperature (Wang et al., 2018), further evidence that translation rates impact Fc‐fusion product quality.

Direct manipulation of the codon sequence was implemented to influence the levels of recombinant protein achieved during culture. This process is typically performed to generate an “optimized” sequence using the most abundant or often used codons for the host species. This, however, does not take into account the availability of the tRNA population for subsequent translation of the sequence. tRNA abundance has been shown to influence translation fidelity (Kapur & Ackerman, 2018), and thus, it is not just codon usage that is relevant, but also the available tRNA population that should be considered. By manipulating the codons used in the Fc‐fusion transcript we showed that both overall expression and fragmentation could be “tuned.” Thus, for such Fc‐fusion proteins, it may not be the “fastest” decoding sequence that is optimal in terms of the balance between intact product yield and fragmentation. These data collectively suggest that the approach of optimizing the gene codon sequence for Fc‐fusion transcripts prone to fragmentation to achieve high rates of translation may not always be beneficial to the yield and quality of the final product obtained in agreement with previous suggestions (Zhou et al., 2013).

**Figure 7:**
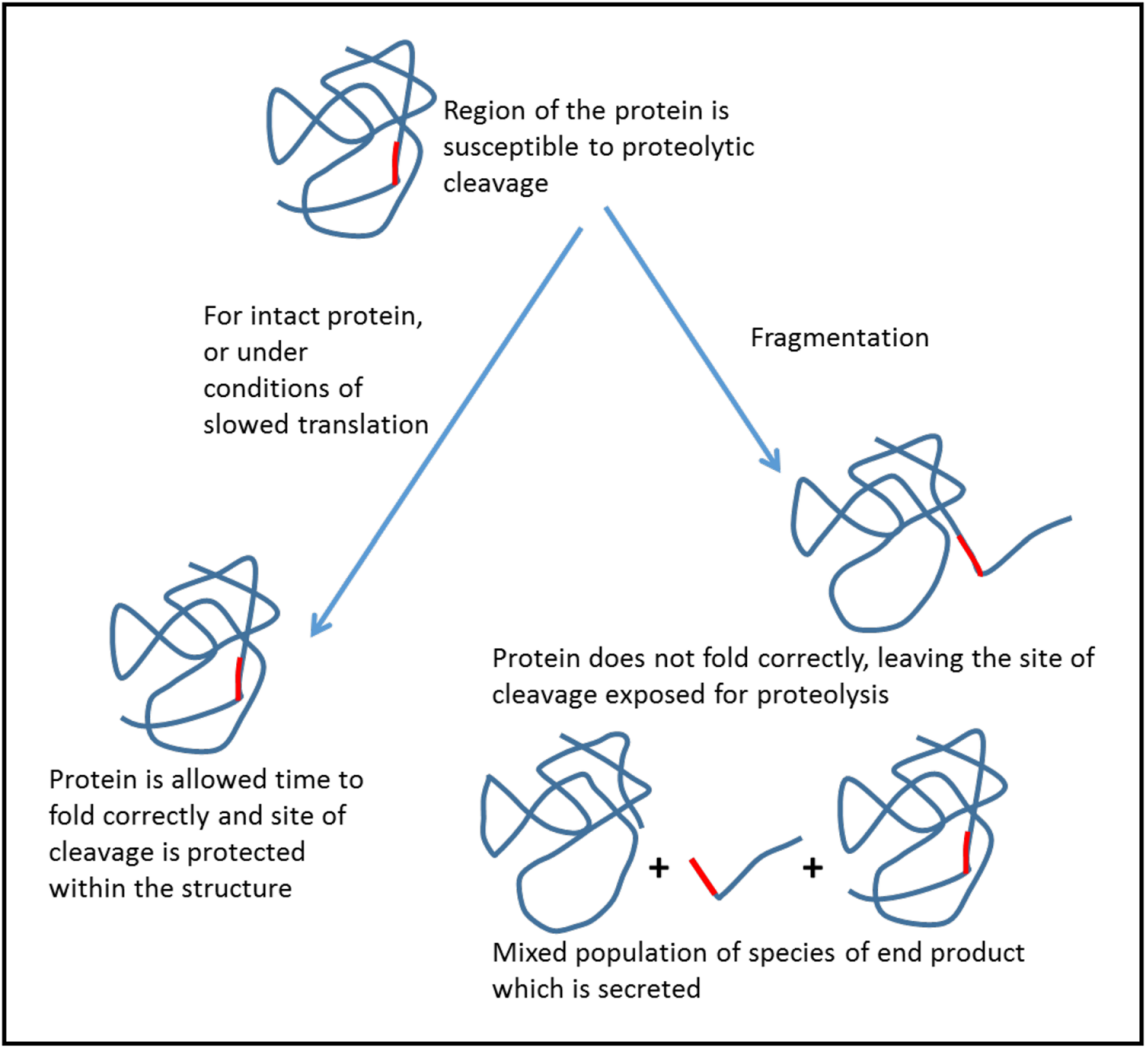
Proposed hypothesis for the reduced fragmentation of the immunoglobulin G1 Fc‐fusion molecule upon slowed messenger RNA translation elongation

High rates of elongation can result in folding errors and decreased fidelity, generating a region susceptible to degradation/proteolysis (Sherman & Qian, 2013; Spencer et al., 2012; Xie et al., 2019). As improved fidelity of recombinant proteins from CHO cells has been reported with reduced culture temperatures (Masterton et al., 2010), we propose that the slowing of mRNA translation improves the folding of the Fc‐fusion product, reducing those molecules generated that are prone to fragmentation. Incorrect folding, at higher translation elongation and, thus, polypeptide synthesis rates, may result in the exposure of a region of the polypeptide with a cleavage/fragmentation site that is “hidden” when the molecule is “correctly” folded. Other reports in the literature support this whereby cell lines with higher proteolytic cleavage or “clipping” had enriched UPR induction and protein folding capacity over those with lower clipping tendency, suggesting that the fragmentation susceptibility was the result of reduced protein folding efficiency (Henry et al., 2018). This hypothesis is outlined schematically in Figure 7, showing that reduced translation elongation may facilitate improved folding and therefore reduced susceptibility to proteolytic attack. An alternative explanation, although with less evidence, is that fast elongation and slow secretion increases the intracellular pool of Fc‐fusion protein present making proteolysis more likely.

In summary, the impact of tuned mRNA translation, particularly elongation, in the production of the model IgG1 Fc‐fusion protein investigated here shows that this is an approach that can be investigated to mitigate against fragmentation and “titrate” the balance of intact product compared to the clipped product during production. These principles can potentially be applied to other molecules that are difficult to express due to potential folding or fragmentation/clipping issues. A small decrease in the desired product yield can be outweighed by a reduction in the heterogeneous population obtained, making downstream processing less challenging where significant loss of the desired product may occur when large amounts of impurity product (e.g., fragmented) are present. Overall, the key message from this study is that “fastest is not always best” and there are sometimes clear benefits to the tailoring of translation elongation to produce the “best” quality yields from a bioprocess rather than the “highest” yields.

## AUTHOR CONTRIBUTIONS

**Tanya J. Knight**: Conceptualization, investigation, methodology, writing–original draft, writing–review & editing. **Jane F. Povey**: Investigation, methodology, writing–review & editing. **Davide Vito**: Methodology, writing–review & editing. **Atul Mohindra**: Conceptualization, funding acquisition, supervision, writing–review & editing. **Colin M. Jaques**: Conceptualization, funding acquisition, methodology, supervision, writing–review & editing. **C. Mark Smales**: Conceptualization, funding acquisition, methodology, supervision, writing–original draft, writing–review & editing.

## CONFLICTS OF INTEREST

Atul Mohindra and Colin M. Jaques are employed by Lonza Biologics. Lonza Biologics owns and licenses the GS Gene Expression System®, CHOK1SV®, and CHOK1SV GS‐KO® cell lines. The remaining authors declare no conflict of interest.

## Supporting information

Supporting information.

## Data Availability

The data that support the findings of this study are available from the corresponding author upon reasonable request.
