# Supplementary figures and images for: Manipulation of mRNA translation elongation influences the fragmentation of a biotherapeutic Fc‐fusion protein produced in CHO cells
Source: Biotechnol Bioeng. 2022 Sep 18;119(12):3408–20. doi: 10.1002/bit.28230 (PMC9826484; doi:10.1002/bit.28230)

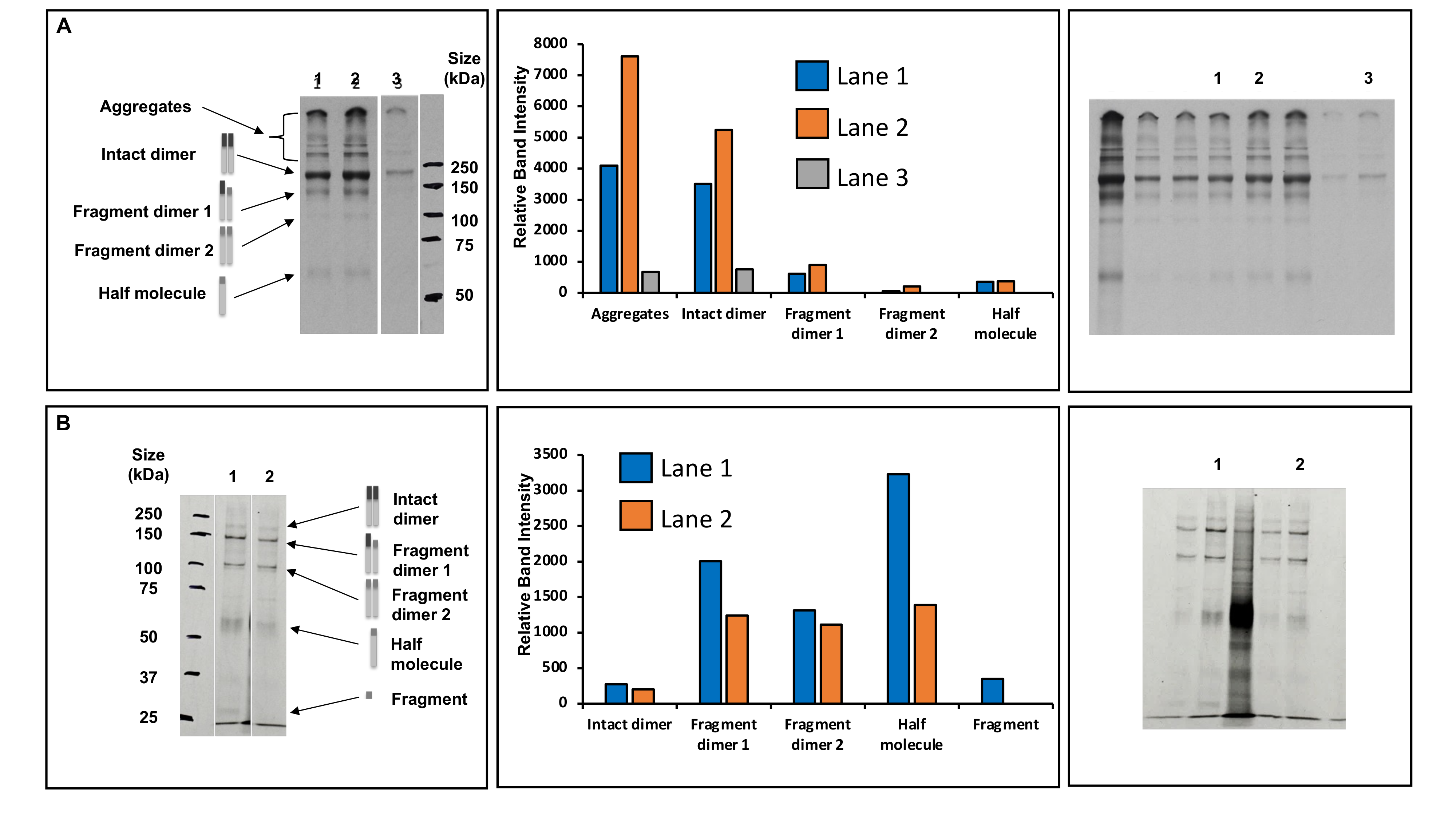

Supplement: Supplementary file 1 — Supporting information. [file BIT-119-3408-s001.tif]
